# The effectiveness of hybrid treatment for sever multiple trauma: a case of multiple trauma for damage control laparotomy and thoracic endovascular repair

**DOI:** 10.1186/s12245-017-0145-8

**Published:** 2017-06-05

**Authors:** Naofumi Bunya, Keisuke Harada, Yosuke Kuroda, Tsubasa Toyohara, Takashi Toyohara, Narumi Kubota, Ryuichiro Kakizaki, Hideto Irifune, Shuji Uemura, Eichi Narimatsu

**Affiliations:** 1 Department of Emergency Medicine, Sapporo Medical University, S1W16 Chuo-ku, Sapporo, Hokkaido 060-8543 Japan; 2 Department of Cardiovascular Surgery, Sapporo Medical University, S1W16 Chuo-ku, Sapporo, Hokkaido 060-8543 Japan

**Keywords:** Hybrid treatment, REBOA, Damage control surgery, TEVAR, Interventional radiology

## Abstract

**Background:**

Time is a crucial factor for the successful early management of the multi-trauma patient. Hybrid operating theaters, which support the integration of surgical treatment and interventional radiology, provide opportunities to reduce the time-to-surgery for life threatening conditions.

**Case presentation:**

We describe the early successful treatment of a 54-year-old male who sustained multiple injuries when he was hit by a 1000 kg bale of wheat that fell from a height. He was admitted with hemorrhagic shock due to intra-abdominal bleeding, an unstable fracture of the pelvis, and blunt aortic injury, which was considered to be at high risk of rupture. External fixation was applied to the pelvis in the resuscitation bay, and the patient was transferred to a hybrid operating theater for treatment of both the intra-abdominal hemorrhage and blunt aortic injury. Damage control laparotomy and thoracic endovascular aortic repair were performed uneventfully.

**Conclusions:**

Hybrid treatment, which combines emergency surgery and intraoperative interventional radiology, provides a prompt and appropriate management approach for the treatment of patients with severe multiple trauma and may improve patient outcomes.

## Background

Time is a crucial factor for successful early trauma management in critical settings. Once injuries that affect physiological status are identified, emergency treatment, such as damage control surgery (DCS), interventional radiology (IVR), and decompressive craniotomy, must be prioritized. However, as IVR and surgical intervention can only be performed in angiography and operating theaters, respectively, time is needed to transfer patients, who are medically unstable, from the operating room to the angiography suite. Hybrid operating theaters eliminate this need by integrating fixed high-quality angiography equipment within the surgical environment. As such, hybrid operating theaters are increasingly becoming available worldwide and could improve the survival rate of severe multi-trauma victims [1–3]. In this case report, we describe our use of a hybrid treatment approach, namely, damage control laparotomy and thoracic endovascular aortic repair (TEVAR), for the resuscitative management of a 54-year-old male who presented with multiple trauma.

## Case presentation

The 54-year-old male patient sustained multiple injuries when a 1000 kg bale of wheat fell on him from a height. On initial survey by the emergency medical service, the patient reported severe chest and back pain. His vital signs were as follows: a respiratory rate of 40 breaths/min, heart rate of 140 beats/min and blood pressure of 71/58 mmHg. The patient was transported to our hospital by helicopter. During transport, the emergency physician observed a right thoracic subcutaneous emphysema, bilateral deformation of the thoracic wall, tenderness over the pelvis, and a right upper arm deformity. An intravenous line was placed to initiate fluid infusion, and a thoracostomy was performed with placement of a drainage tube for a suspected right pneumothorax.

Upon admission to our resuscitation bay, the patient’s initial vital signs were as follows: Glasgow coma scale 13 (eye opening response, 4; verbal response, 4; motor response, 5); systolic blood pressure, 68 mmHg; heart rate, 147 beats/min; respiratory rate, 33 breaths/min; and pulse oximetry of 92%, under a reservoir mask oxygen flow of 12 L/min. On examination, a right thoracic subcutaneous emphysema, decreased breath sounds, bilateral deformation of the rib cage, and an unstable pelvic ring fracture were revealed. Based on our emergency protocol, the patient was intubated, a left chest tube was inserted and transfusion was initiated.

**Fig. 1:**
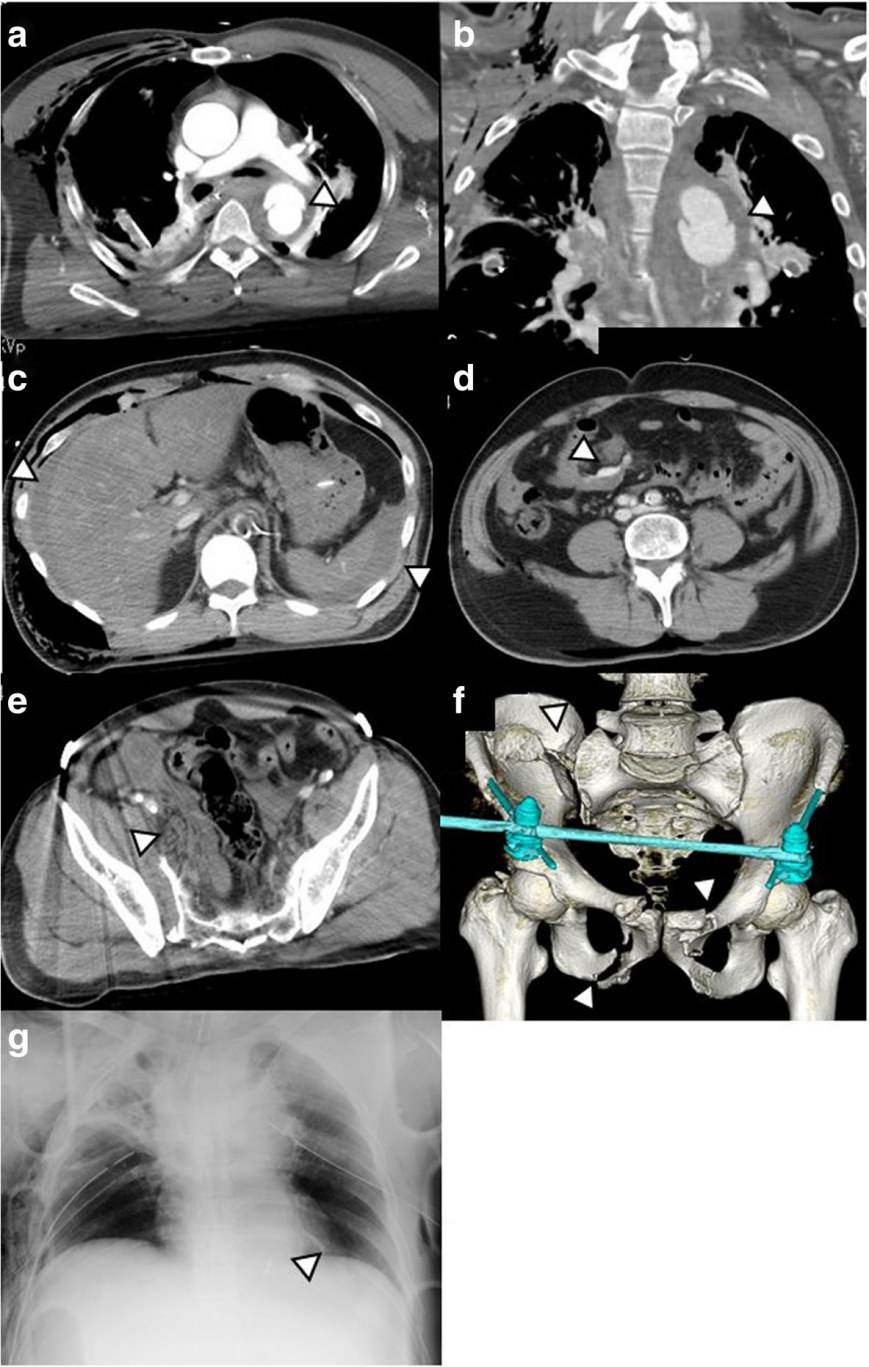
Chest radiography and computed tomography on initial resuscitation. **a**, **b** Thoracic injury to the aorta. **c** Hemoperitoneum. **d** Extravasation from the mesenteric injury. **e** Retroperitoneum from the pelvic fracture. **f** Fracture of the pelvis shown from reconstructed computed tomography images, and **g** position of resuscitative endovascular balloon-occlusion of the aorta (REBOA), with the *marker* indicating the top of the REBOA

**Fig. 2:**
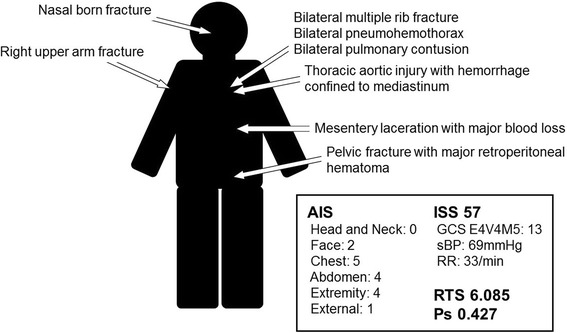
Clinical scores at admission, including the abbreviated injury score (*AIS*), injury severity score (*ISS*), revised trauma score (*RTS*), and the probability of survival (*Ps*)

Radiographic assessment identified an open-book type pelvic fracture, with apparent fluid collection, which was not evident during the initial ultrasound examination due to the poor imaging quality caused by the subcutaneous emphysema. A resuscitative endovascular balloon occlusion of the aorta (REBOA) was placed to control the intra-abdominal hemorrhage, and external fixation for the pelvic fracture was applied. Subsequent contrast computed tomography (CT) imaging revealed the following multiple trauma: blunt aortic injury at the isthmus; bilateral hemopneumothorax; multiple bilateral rib fractures; pulmonary contusions; mesenteric injury with intra-abdominal hemorrhage; pelvic fracture with retroperitoneal hemorrhage, without extravasation; and a fracture of the nasal bone (Figs. 1 and 2). Based on the CT imaging results, the following prioritized treatment strategy was implemented. As the patient’s hemodynamic status depended on inflation of the REBOA, control of the intra-abdominal hemorrhage by DCS was prioritized. In addition, the blunt injury to the aorta was considered to be at high risk of rupture, due to the presence of a large pseudoaneurysm meeting the criteria for high risk of rupture, including a >50% of the aortic circumference, in combination with a mediastinum hematoma [4], which required immediate treatment. To allow us to effectively manage both of these priorities in a continuous manner, we selected to transfer the patient to the hybrid operating theater in our facility which has the capacity for both laparotomy and TEVAR.

**Fig. 3:**
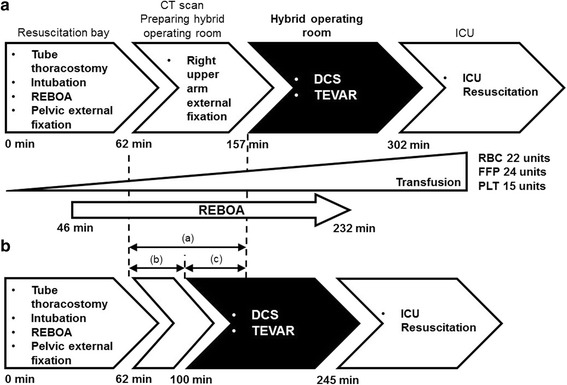
Timeline of trauma resuscitation for our case compared to an ideal resuscitation timeline. **a** Timeline of trauma resuscitation for our case, indicating the wait time between computed tomography imaging and completion of the preparation of the operative theater; during this wait time, external fixation was applied to stabilize the fracture of the right arm. **b** An ideal timeline of trauma resuscitation, indicating that time-to-surgery could have been shortened by 1 h if the hybrid operating theater had been promptly available, which would also have reduced the volume of transfusion required by reducing the time-to-inflation of the resuscitative endovascular balloon occlusion of the aorta (*REBOA*). Comparison of the real and ideal timeline of resuscitative trauma, showing: (a) real time for preparation of the hybrid operating theater, 95 min; (b) simulation, showing that the hybrid operating theater can be prepared in 40 min; and (c) simulation showing that the time-to-surgery can be reduced by approximately 1 h. Abbreviations: *DCS* damage control surgery, *TEVAR* thoracic endovascular repair, *REBOA* resuscitative endovascular balloon occlusion of the aorta, *CT* computed tomography, *RBC* red blood cell, *FFP* fresh frozen plasm, *PLT* platelet

On laparotomy, we observed a mesenteric injury, associated with a 2.5 L hemoperitoneum. Due to the extent of injury, suture of the site was performed using an open abdominal management. Following the surgical procedure, inflation of the REBOA was no longer necessary to control the bleeding. We subsequently performed the TEVAR, which was completed without adverse in about 30 min (Fig. 3a). The open abdominal surgical incision was closed at day 2 post-admission, and internal fixation of the fracture of the pelvis was performed on day 7. The patient was extubated on day 12. He was transferred to another hospital for rehabilitation 1 month post-trauma, with ambulatory discharge at 4 months post-trauma.

## Discussion

Hybrid operating theaters provide two distinct advantages for the treatment of patients with severe multi-trauma: integration of IVR to surgical treatment, which shortens the time-to-treatment of critical injuries [2, 3], and elimination of the need to transfer patients from the trauma bay to the operating room and angiography suite [2, 3]. The patient in our case report presented with three life-threatening injuries that required prompt intervention: aortic injury, with pseudoaneurysm >50% of the aortic circumference along with a mediastinum hematoma which are indicative of a high risk for rupture [4], a massive hemoperitoneum due to mesentery laceration, and an unstable pelvic fracture. Our initial treatment included application of external fixation for the unstable pelvic fracture and insertion of the REBOA in the resuscitation bay. We then proceeded with treatment of the other two fatal injuries, selecting to use the hybrid operating room due to the extent of those injuries. As this was our first experiencing using the hybrid operating room for the treatment of a patient with multiple trauma, we required extra preparation time (Fig. 3a). While we used REBOA to provide initial hemodynamic control [5], the hybrid operating theater allowed use to directly control intra-abdominal bleeding and perform TEVAR in a continuous manner, without the need for further transfer of our patient, which likely contributed to saving our patient’s life by reducing the time-to-surgery. In Fig. 3b, we demonstrate that we could have further reduced the time-to-surgery by about 1 h with prior experience in managing the hybrid operating room environment. Effective use of a hybrid operating theater for multiple trauma requires training using simulation involving the entire multidisciplinary team [3].

## Conclusions

Hybrid treatment, which combines emergency surgery and IVR, provides a prompt and appropriate management approach for the treatment of patients with severe multiple trauma and may improve patient outcomes. Simulation training for the entire multidisciplinary trauma team can improve efficiency and can shorten the time-to-surgery to manage life threatening conditions.

## Abbreviations

CT: Computed tomography
DCS: Damage control surgery
IVR: Interventional radiology
REBOA: Resuscitative endovascular balloon occlusion of the aorta
TEVAR: Thoracic endovascular aortic repair
